# Supplementary material for: Fluorometric Detection of Thiamine Based on Hemoglobin–Cu3(PO4)2 Nanoflowers (NFs) with Peroxidase Mimetic Activity
Source: Sensors (Basel). 2020 Nov 7;20(21):6359. doi: 10.3390/s20216359 (PMC7664642; doi:10.3390/s20216359)
Supplement: Supplementary file 1 [file sensors-20-06359-s001.pdf]

Supplementary Materials

Fluorometric Detection of Thiamine Based on Hemoglobin-$Cu_3(PO_4)_2$ Nanoflowers with Peroxidase Mimetic Activity

Hangjin Zou, Yang Zhang, Chuhan Zhang, Rongtian Sheng, Xinming Zhang, Yanfei Qi*

School of Public Health, Jilin University, Changchun, Jilin 130021, China.

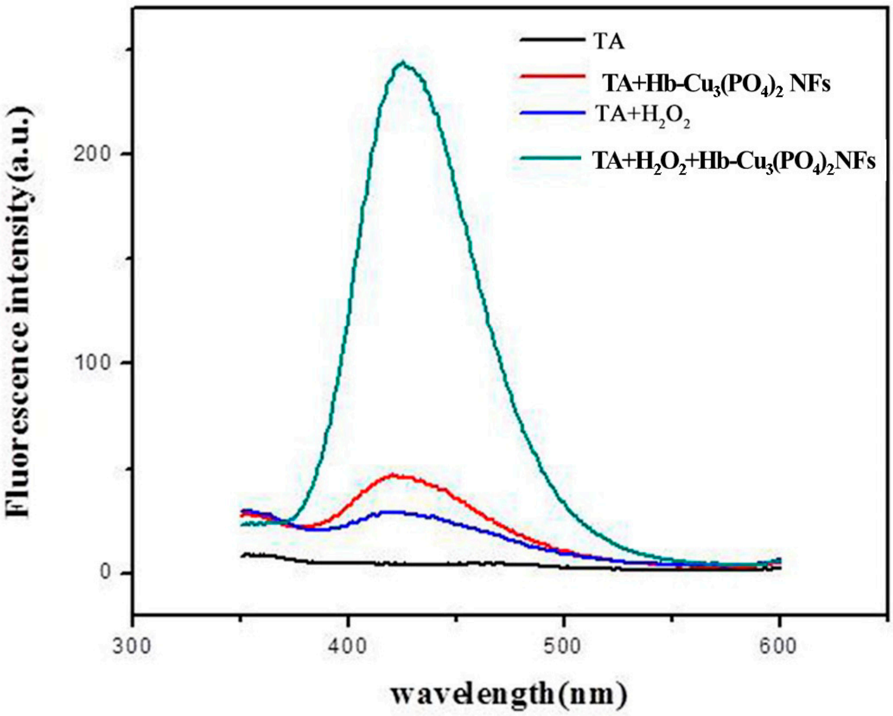

Figure. S1 Fluorescence spectra for detection of hydroxyl radicals ·OH with different controls. Reaction conditions: 100 mM H_2O_2, pH 7.4, 25 mM TA, 2 mg/mL Hb–$Cu_3(PO_4)_2$ NFs, temperature 55 °C, incubation time 60 min.

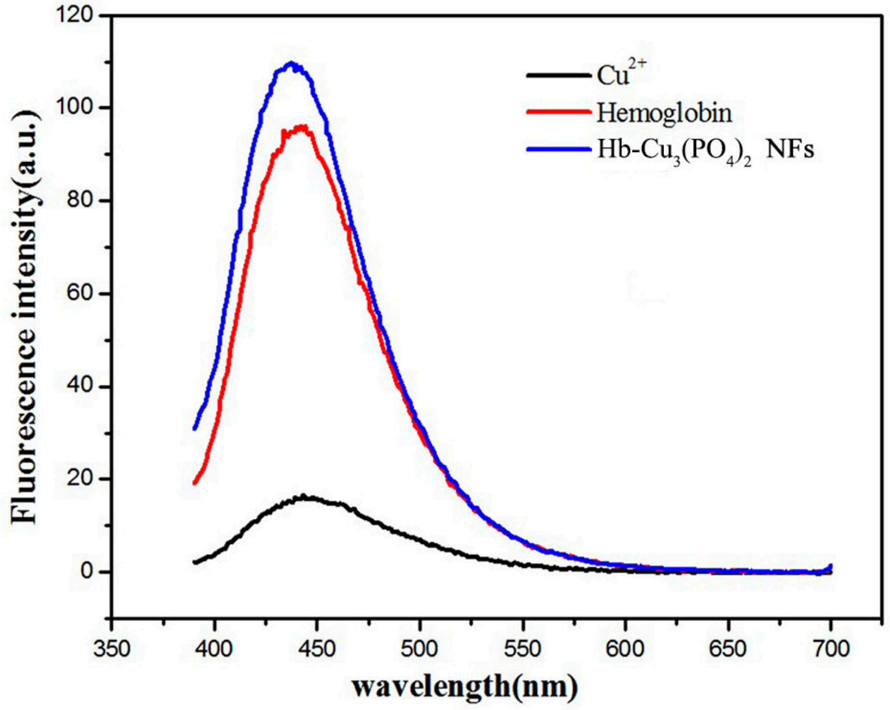

Figure. S2 The fluorescence intensities of thiochrome in the catalytic activities of $CuSO_4$, hemoglobin and Hb–$Cu_3(PO_4)_2$ NFs with the same concentration of 1 mg/mL. Reaction conditions: 1.25 mM H_2O_2, 1 mM TH at pH 12 buffer solution, incubation time 5 min, 25 °C.

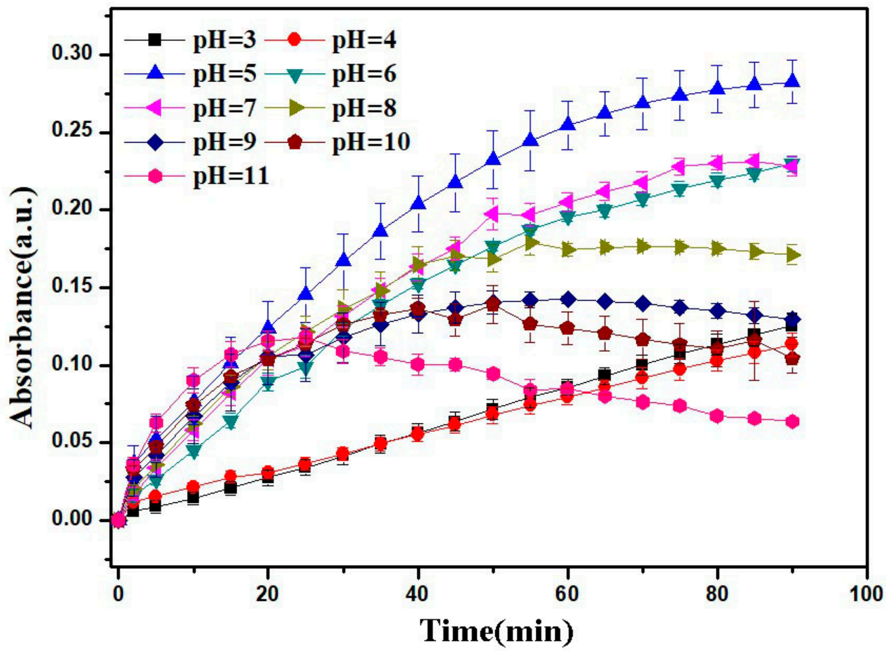

Figure. S3. The pH Dependency with catalytic activity of Hb–$Cu_3(PO_4)_2$ NFs using pH 3–11. Reaction conditions: 100 mM H_2O_2, 2 mM OPD, 2 mg/mL Hb–$Cu_3(PO_4)_2$ NFs, temperature 37 °C.

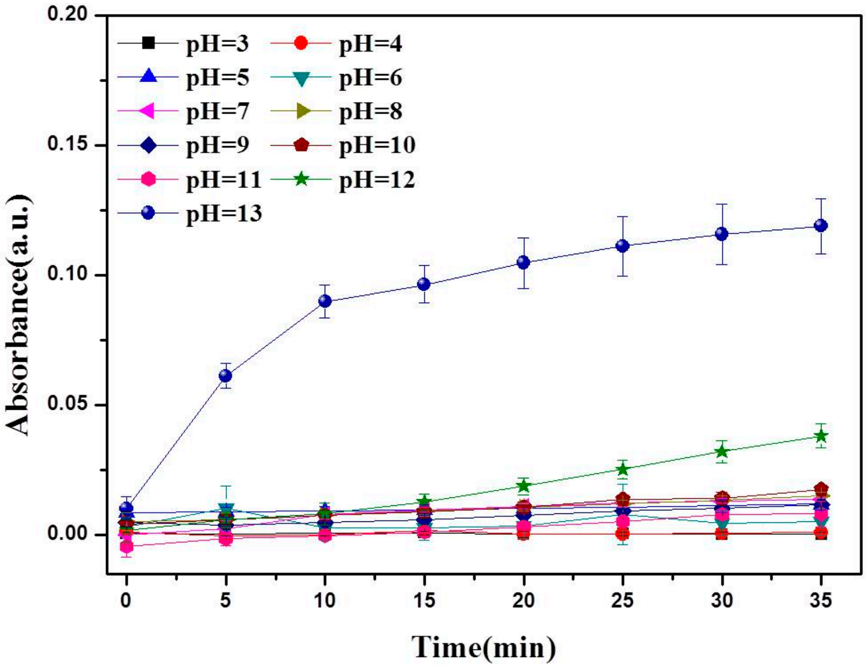

Figure. S4. The pH Dependency with oxidase activity of Hb–$Cu_3(PO_4)_2$ NFs. Reaction conditions: 2 mM OPD, 2 mg/mL Hb–$Cu_3(PO_4)_2$ NFs, temperature 37 °C.

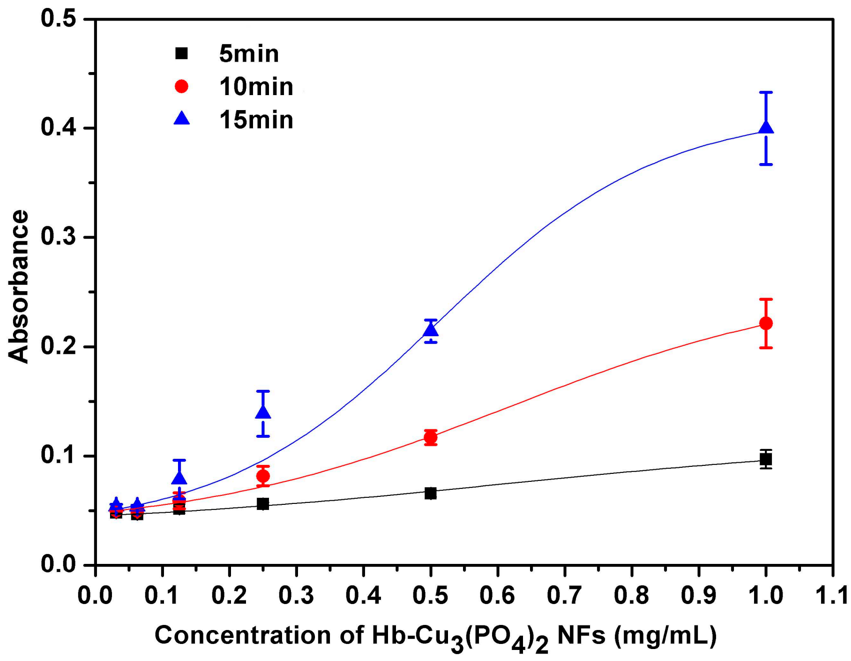

Figure. S5. Dependency of OPD oxidation activity on the Hb–$Cu_3(PO_4)_2$ NFs concentration (0.03125–1mg/mL) and time(5–15min). The rate of the reaction was determined by monitoring the increase of absorbance at 450 nm, which is related to the formation of DAP, in the presence of 100 mM of H_2O_2 and 2 mM OPD at pH 10, 37 °C.

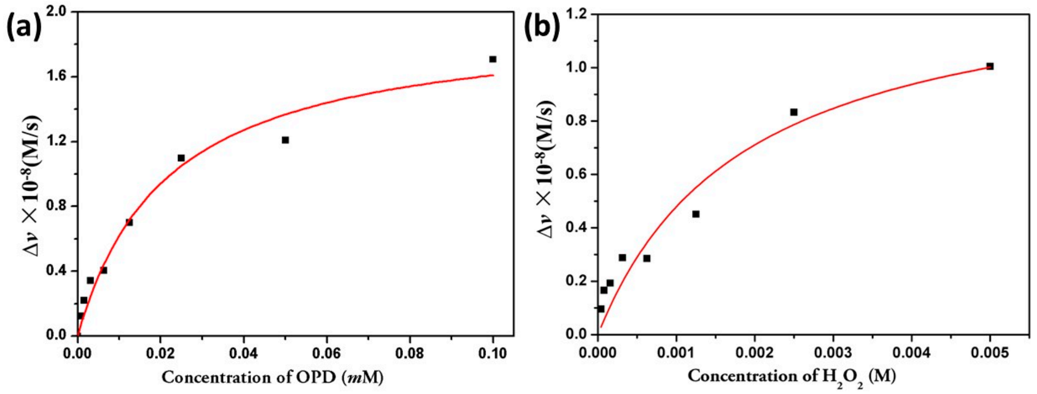

Figure. S6. Steady-state kinetic study of Hb–$Cu_3(PO_4)_2$ NFs. Michaelis-Menten curve for (**a**) variation of OPD concentration (0–2 mM) and keeping the H_2O_2 (0.1 M) and Hb–$Cu_3(PO_4)_2$ NFs (2 mg/mL) concentration constant and (**b**) variation of H_2O_2 concentration (0–0.1M) and maintaining OPD (2 mM) and Hb–$Cu_3(PO_4)_2$ NFs (2 mg/mL) concentration constant. The formation of OPD at 450 nm was monitored for 300 s at 37 °C.
